# Dietary 5-aminolevulinic acid modulates gut microbiota, reduces oxidative stress, and enhances immune status in weanling piglets: An 8-week exploratory study

**DOI:** 10.14202/vetworld.2026.355-365

**Published:** 2026-01-30

**Authors:** Shodai Ishikawa, Kiyonori Kawasaki², Kiminobu Yano, Kimiko Kazumura, Takamitsu Tsukahara, Shin Taniguchi, Hiroto Miura, Ryo Inoue

**Affiliations:** 1 Laboratory of Animal Science, Setsunan University, Hirakata, Osaka 573-0101, Japan; 2 Graduate School of Agriculture, Kagawa University, Kita, Kagawa 761-0795, Japan; 3 Global Strategic Challenge Center, Hamamatsu Photonics K.K., 5000 Hirakuchi, Hamana-ku, Hamamatsu, Shizuoka 434-8601, Japan; 4 Kyoto Institute of Nutrition & Pathology, Tsuzuki, Kyoto 610-0231, Japan; 5 Department of Animal Risk Management, Faculty of Risk and Crisis Management, Chiba Institute of Science, Choshi, Chiba 288-0025, Japan

**Keywords:** 5-aminolevulinic acid, antioxidant capacity, gut microbiota, immune response, piglets, oxidative stress, weaning stress, swine nutrition

## Abstract

**Background and Aim:**

Five-aminolevulinic acid (5-ALA), a precursor in heme biosynthesis, has gained attention as a functional feed additive due to its reported benefits on metabolism, redox balance, and immunity. Although supplementation in sows and broilers has demonstrated favorable physiological outcomes, its effects on the gut microbiota and immune–oxidative profiles of weanling piglets remain unclear. This study aimed to evaluate whether 5-ALA supplementation modifies fecal microbiota composition and influences oxidative stress and immune parameters in piglets during the post-weaning period.

**Materials and Methods:**

Twelve 28-day-old piglets were randomly allocated to a control or 5-ALA group (20 mg/kg feed) for 56 days. Body weight (BW), fecal samples, and blood samples were collected at 28, 56, and 84 days of age. Fecal microbiota was characterized by 16S rRNA sequencing (QIIME2). Oxidative stress and inflammatory markers in leukocytes (superoxide radical, hypochlorite ion) were quantified using a dual chemiluminescence/fluorescence system. Plasma malondialdehyde (MDA) and immunoglobulin G (IgG) were measured to assess systemic oxidative damage and humoral immunity. Statistical analyses included Permutational Multivariate Analysis of Variance, LEfSe, and Spearman correlations.

**Results:**

BW did not differ significantly between groups, although the 5-ALA group tended to be heavier at 56 days. β-diversity differed significantly between groups at 56 and 84 days. Six and eight bacterial genera were differentially abundant at 56 and 84 days, respectively; 5-ALA supplementation enriched short-chain fatty acids-associated genera such as *Coprococcus*, *Prevotellaceae* UCG-003, and *Phascolarctobacterium*. At 84 days, the 5-ALA group showed markedly lower leukocyte superoxide levels (~3.5-fold reduction; p < 0.05) and a tendency toward lower hypochlorite ion production. Plasma IgG concentration was approximately 1.5-fold higher in the 5-ALA group (p < 0.05). Multiple bacterial genera exhibited significant correlations with oxidative and immune markers.

**Conclusion:**

Dietary supplementation with 5-ALA altered the fecal microbiota and improved oxidative and immune status in weanling piglets, suggesting functional modulation of the gut–immune axis. Although exploratory and based on a small cohort, the findings warrant further controlled studies to validate dose–response effects and elucidate mechanistic pathways.

## INTRODUCTION

Five-aminolevulinic acid (5-ALA) is a naturally occurring amino acid and a precursor of protoporphyrin IX, the fundamental building block of heme [1]. When ferrous iron is inserted into the center of protoporphyrin IX, the molecule is converted into heme, an essential cofactor for numerous proteins involved in cellular metabolism [2]. Heme-dependent enzymes, including cytochrome c oxidase (Complex IV), play critical roles in mitochondrial oxidative phosphorylation and adenosine triphosphate generation [3]. Because the heme biosynthetic pathway tightly regulates metabolic flux through enzymes such as ALA dehydratase and porphobilinogen deaminase, changes in ALA availability can influence mitochondrial activity and cellular redox balance. Excess accumulation of porphyrin intermediates may increase reactive oxygen species production, placing a greater demand on antioxidant systems to maintain homeostasis [4].

Interest in 5-ALA as a functional feed additive for livestock has grown since around 2010, primarily due to its reported benefits on immunity, metabolic function, and overall health [5]. For instance, dietary 5-ALA supplementation in Holstein cows has been associated with increased milk protein content [6], and broiler chickens supplemented with 5-ALA exhibit improved resilience to lipopolysaccharide-induced oxidative and inflammatory stress [7].

In swine, supplementation with 5-ALA has been shown to improve iron status in sows [8] and increase birth weight in their offspring [9]. Studies in weanling piglets further suggest immunomodulatory effects, including elevated proportions of CD8+ T cells and B cells in peripheral blood [10]. Collectively, these findings indicate that 5-ALA may enhance immune competence and disease resistance in young pigs.

The gut microbiota, a diverse and dynamic community of 500–1,000 bacterial species residing in the digestive tract, plays an essential role in host health, nutrient utilization, and disease prevention [11, 12]. In modern pig production, gut microbiota composition has become a major focus due to its strong association with productivity and feed efficiency [13]. Greater microbial diversity is generally linked to improved digestion and nutrient absorption [14].

Weaning represents one of the most physiologically stressful periods in a pig’s life. Abrupt dietary, environmental, and social changes at weaning induce marked alterations in the intestinal environment, including villous atrophy [15] and substantial shifts in microbiota composition [16]. Such weaning-associated dysbiosis can impair growth performance [17] and disrupt immune system maturation [18], highlighting the importance of strategies that help stabilize the gut microbiota during this period.

Although 5-ALA supplementation in sows has been shown to modify maternal gut microbiota [19], its effects on the gut microbiota of nursery and growing piglets remain largely unexplored.

Although 5-ALA has been investigated as a promising feed additive for enhancing metabolic function, oxidative balance, and immune responsiveness in livestock, its effects on the gut microbiota of piglets remain poorly understood. Existing studies have focused primarily on sows or on systemic physiological responses in piglets, without evaluating how 5-ALA may reshape the microbial ecosystem during the critical post-weaning period, a time marked by instability in gut microbial structure and heightened susceptibility to oxidative and immunological stress. Moreover, no previous work has simultaneously assessed microbiota composition alongside oxidative and immune markers in weanling pigs, despite the well-established interaction between the gut microbiota, redox state, and immune function. Consequently, the mechanistic link between 5-ALA supplementation, microbial modulation, and host physiological outcomes in nursery piglets has not been elucidated. This lack of integrated data limits our understanding of whether 5-ALA acts directly or indirectly on the gut–immune axis and whether such effects have practical implications for piglet health and development.

To address this gap, the present study conducted an 8-week exploratory feeding trial to evaluate the effects of dietary 5-ALA supplementation on weanling piglets. Specifically, we aimed to (i) characterize changes in the fecal microbiota using *16S rRNA* gene sequencing, (ii) assess alterations in oxidative and inflammatory status in leukocytes, and (iii) determine whether 5-ALA influences systemic immunity through measurements of plasma immunoglobulin (Ig)G. By integrating microbial, oxidative, and immunological datasets, this study sought to generate hypothesis-forming evidence regarding the potential of 5-ALA to modulate the gut–immune axis during the vulnerable post-weaning phase. The findings are intended to provide foundational insights for future mechanistic and dose–response studies aimed at optimizing 5-ALA use in pig production.

## MATERIALS AND METHODS

### Ethical approval

All procedures involving animals were reviewed and approved by Animal Experiment Committee of Kagawa University, Japan (approval No. 22647; approval date: 23 August 2022). The study was conducted in accordance with the Act on Welfare and Management of Animals of Japan and the institutional guidelines for the care and use of laboratory/experimental animals, and it complied with internationally accepted principles for animal research (Replacement, Reduction, and Refinement). The trial was designed as an 8-week exploratory randomized controlled feeding study in weaned piglets, and the minimum number of animals required to address the study objectives was used. Piglets were monitored at least daily by trained personnel for general health, behavior, and feed and water intake, with enhanced monitoring during the immediate post-weaning period. Housing, handling, and sampling procedures were implemented to minimize pain and distress; blood collection from the jugular vein was performed by experienced staff using sterile equipment and appropriate restraint to reduce handling time. Humane endpoints were predefined before study initiation, including but not limited to severe lethargy, persistent anorexia, dehydration, uncontrolled diarrhea, severe lameness, respiratory distress, or body condition deterioration; any animal meeting endpoint criteria would have received veterinary assessment and, if necessary, been withdrawn from the study and humanely managed according to institutional policy. No surgical procedures were performed, and no euthanasia was planned as part of the protocol; if euthanasia became necessary for welfare reasons, it would have been carried out using IACUC-approved methods. The study is reported in accordance with the Animal Research: Reporting of In Vivo Experiments 2.0 guidelines to ensure transparency and reproducibility.

### Study period and location

The study was conducted from August 2022 to February 2025 at the farm and laboratory of Kagawa University and the laboratory of Setsunan University, located in Japan.

### Study design

This work was conducted as an 8-week exploratory randomized controlled feeding trial to investigate the effects of dietary 5-ALA on gut microbiota, oxidative stress, and immune status in weanling piglets.

### Animals and experimental allocation

Twelve weaned piglets (Landrace × Large White × Duroc), 28 days old and originating from three litters, were enrolled. At weaning, piglets weighed 6.21 ± 0.90 kg. Using a stratified randomization approach based on sex and initial body weight (BW), animals were assigned to either the control (n = 6) or 5-ALA group (n = 6), ensuring balanced sex distribution (four castrated males and two females per group).

The 5-ALA group received 20 mg of 5-ALA per kg of feed, top-dressed using 2 g of Mitochon Power® (1% 5-ALA; Bussan Animal Health, Osaka, Japan). Feed was freshly prepared daily to prevent degradation. Water and feed were provided ad libitum.

### Diet formulation and nutrient composition

All diets were provided in mashed form and consisted of commercial prestarter (Winny Z), starter (Gattsuku Milk), grower (Nexcel Milk Stage C), and developer (Winny B) formulations from Nihon Nosan Kogyo Co., Ltd. (Yokohama, Japan). Diets met or exceeded nutrient requirements defined by the Japanese Feeding Standard for Swine. Guaranteed nutrient values were:

1. Prestarter: CP ≥ 24.5%, EE ≥ 6.0%, CF ≤ 2.0%, ash ≤ 7.0%, Ca ≥ 0.75%, p ≥ 0.65%
2. Starter: CP ≥ 21.0%, EE ≥ 4.5%, CF ≤ 2.0%, ash ≤ 8.0%, Ca ≥ 0.65%, p ≥ 0.55%
3. Grower: CP ≥ 20.0%, EE ≥ 4.0%, CF ≤ 3.0%, ash ≤ 7.0%, Ca ≥ 0.60%, p ≥ 0.50%
4. Developer: CP ≥ 18.5%, EE ≥ 3.5%, CF ≤ 4.0%, ash ≤ 6.5%, Ca ≥ 0.65%, p ≥ 0.50%

No amino acid or metabolizable energy values were provided by the manufacturer. No supplements other than 5-ALA were added. Proximate composition analyses were performed in-house, and results are presented in Table 1.

**Table 1:** Proximate nutrient composition of experimental diets fed to weanling piglets.

Parameter (%)	Pre-starter	Starter	Grower	Developer
Moisture	7.35	7.01	8.62	7.38
Crude protein	28.76	26.61	24.75	21.48
Crude fiber	0.40	0.45	1.01	2.05
Ether extract	6.44	6.86	7.64	7.17
Ash	7.08	6.44	6.66	6.66
Ca	0.83	0.69	0.61	0.74
P	0.73	0.83	0.78	0.65

Values represent the median of duplicate laboratory measurements. All nutrient contents are expressed as percentages on an as-fed basis. No additional supplements other than dietary 5-aminolevulinic acid were included. CP = Crude protein, CF = Crude fiber, EE = Ether extract, Ca = Calcium, P = Phosphorus.

Piglets received prestarter feed during week 1, starter feed in week 2, grower feed for weeks 3–5, and developer feed for weeks 6–8.

### Housing, management, and sample collection

Piglets were housed in conventional nursery pens (1,800 cm × 2,100 cm) under standard farm conditions. Mean ambient temperature and humidity were 22.6°C ± 4.1°C and 78.8% ± 8.7%, respectively. Feed intake was recorded daily at the pen-level, whereas physiological and microbiota measurements were collected individually.

BW, blood, and feces were sampled at 28, 56, and 84 days of age. Blood was collected from the jugular vein using sterile 21-gauge needles into heparinized tubes (Terumo, Osaka, Japan). Fresh feces were collected into sterile 15-mL tubes. Whole blood (3 µL) was used immediately for oxidative and inflammatory assays, while plasma and feces were stored at −80°C until analysis.

### *16S rRNA* gene sequencing and microbiota analysis

Genomic DNA was extracted from fecal samples using the QuickGene DNA Tissue Kit SII (Kurabo, Osaka, Japan). DNA quantity and purity were assessed via Nanophotometer (Implen, Kusatsu, Japan), ensuring A260/280 values of 1.8–2.0.

Library preparation followed Inoue *et al*. [20], including V3–V4 region amplification, dual indexing, and sequencing on the Illumina MiSeq platform. Successful amplification was confirmed using MultiNA electrophoresis (Shimadzu, Kyoto, Japan).

Sequence data processing followed Yoshimura *et al*. [21]. Reads were analyzed in QIIME2 (v.2021.11; https://docs.qiime2.org/2021.11/) [22] using DADA2 for quality control, denoising, and amplicon sequence variant inference. Taxonomic assignment employed the SILVA 138 database with a pre-trained Naïve Bayes classifier (confidence threshold = 0.7). Singletons and sequences classified as chloroplasts or mitochondria were removed. Phylogenetic tree construction used SATé-enabled phylogenetic placement [23]. Alpha and beta diversity indices were computed using standard QIIME2 pipelines.

### Measurement of plasma malondialdehyde (MDA)

Plasma MDA concentrations were quantified using a commercial assay kit (JaICA, Fukuroi, Japan) according to manufacturer protocols.

### Quantification of leukocyte-derived reactive species

Oxidative stress and inflammatory status in whole blood were assessed using a dual chemilumi-nescence/fluorescence system (CFL-H2200; Hamamatsu Photonics, Hamamatsu, Japan) following Kazumura *et al*. [24, 25].

1. Superoxide radical (O_2_•®): measured via chemiluminescence (CL-O_2_•®).
2. Hypochlorite ion (OCl̅): measured via fluorescence (FL-OCl̅), reflecting myeloperoxidase (MPO) activity.

Due to equipment delivery delays, measurements were not performed at day 28. Fresh whole blood was required; frozen samples were unsuitable. Each sample (n = 6 per group) was analyzed once using standard protocols.

### Quantification of plasma IgG

Plasma IgG concentrations were measured using a porcine IgG ELISA kit (Bethyl Laboratories, Montgomery, TX, USA). Samples were diluted 1:500,000, and absorbance was measured at 450 nm using an iMark microplate reader (Bio-Rad, Hercules, CA, USA).

### Statistical analyses

Each piglet served as an independent experimental unit. Normality of BW, CL-O_2_•®, FL-OCl̅, MDA, and IgG was assessed using the Shapiro–Wilk test. Non-normal data were analyzed using the Wilcoxon rank-sum test; otherwise, homoscedasticity was evaluated using the F-test, followed by Student’s or Welch’s t-test where appropriate.

Differences in α-diversity were tested using the Wilcoxon rank-sum test. β-diversity was evaluated via = Permutational Multivariate Analysis of Variance using Bray–Curtis distances. Differential taxonomic abundance was identified using LEfSe (Linear discriminant analysis; LDA score >3.0) [26]. Correlations between bacterial genera and physiological markers (CL-O_2_•®, FL-OCl̅, MDA, and IgG) were assessed using Spearman’s rank correlation.

Significance was set at p < 0.05, with trends recognized at 0.05 ≤ p < 0.10. Statistical analyses and figure generation were conducted in R (v.4.4.2, R Core Team, Vienna, Austria).

## RESULTS

### BW, feed intake, and feed conversion ratio (FCR)

BW changes throughout the 8-week trial are presented in Table 2. At baseline (28 days of age), no significant difference was observed between the control and ALA groups. By 56 days of age, piglets receiving 5-ALA tended to exhibit higher BW than controls. At 84 days of age, although the difference was not statistically significant, the ALA group still showed a numerically greater BW. Overall BW gain across the experimental period did not differ significantly between groups (control: 30.11 ± 1.34 kg/8 weeks; ALA: 32.34 ± 1.69 kg/8 weeks; p = 0.33).

**Table 2:** Body weight, daily feed intake, and feed conversion ratio of piglets in the control and ALA groups.

Parameter	Age	Control	ALA	p-value
Body weight (kg)	28-day-old	6.23 ± 0.19	6.19 ± 0.38	0.938
	56-day-old	15.9 ± 0.43	17.52 ± 0.65	0.095
	84-day-old	36.37 ± 1.3	39.14 ± 1.96	0.294
Daily feed intake(g/day)	28-day-old	800	840	–
	56-day-old	2015	1945	–
Feed conversion rate (g/g)	28-day-old	2.32	2.51	–
	56-day-old	2.76	2.70	–

Body weight values are presented as mean ± standard error. Daily feed intake and feed conversion ratio were recorded at the pen level and are therefore presented as descriptive values only; no statistical comparisons were performed for these parameters. ALA = 5-Aminolevulinic acid, BW = Body weight, FCR = Feed conversion ratio.

Feed intake was recorded at the pen-level, and average daily intake per animal was calculated by dividing total pen intake by the number of piglets housed together. All physiological and microbiota-related measurements were collected individually. FCR was calculated as group-level BW gain divided by total feed intake, with slightly higher FCR values observed in the ALA group throughout the study (Table 2).

### Alpha and beta diversity of the fecal microbiota

The final denoized dataset comprised 1,017,459 high-quality sequences (median = 26,174.5 reads/sample; range: 13,261–44,422). Rarefaction curves for all samples are shown in Supplementary Figure 1.

**Figure 1:**
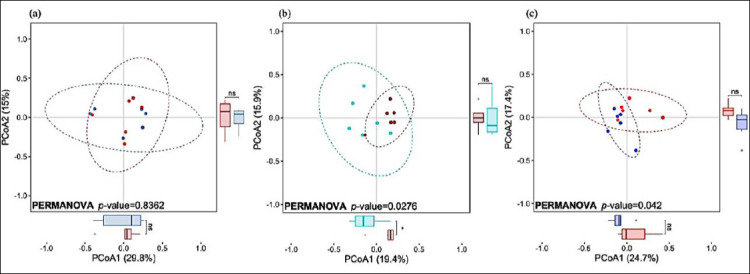
Principal coordinates analysis (PCoA) based on Bray–Curtis distances illustrating fecal microbiota composition in piglets at (a) 28 days, (b) 56 days, and (c) 84 days of age. Samples from the control group are shown in red, whereas samples from the 5-aminolevulinic acid group are shown in blue. Ellipses represent 95% confidence intervals around group centroids. Differences between groups were evaluated using permutational multivariate analysis of variance. An asterisk (*) indicates a significant difference (p < 0.05).

Alpha diversity (Chao1 and Shannon indices) was highest at 56 days of age in both groups but did not differ between the control and ALA groups at any sampling point (Supplementary Table 1; Supplementary Figure 2a).

**Figure 2:**
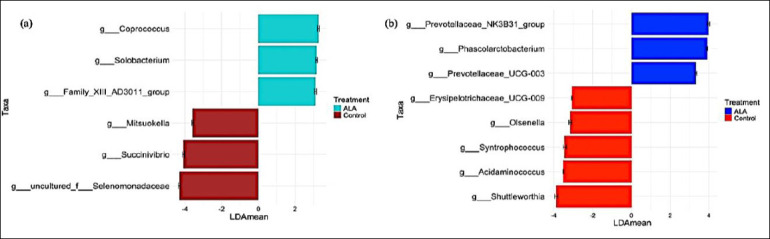
Linear discriminant analysis effect size (LEfSe) identifying bacterial genera that differed significantly between the control and 5-aminolevulinic acid groups at (a) 56 days and (b) 84 days of age. No differentially abundant genera were detected at 28 days of age. Red bars indicate higher relative abundance in the control group, whereas blue bars indicate higher relative abundance in the 5-aminolevulinic acid group.

Beta diversity based on Bray–Curtis distances differed significantly among sampling ages (Supplementary Table 1; Supplementary Figure 2b; p < 0.05). Comparisons between groups revealed significant differences at 56 and 84 days (p < 0.05), but not at 28 days of age (Figure 1).

### Taxonomic differences between control and ALA groups

Substantial temporal shifts in microbial composition were observed across the study. For example, the abundance of *Christensenellaceae R-7* group was highest at 28 days and decreased thereafter, whereas *Prevotella* showed the opposite pattern.

No taxonomic differences were detected between groups at baseline. At 56 days of age, six genera differed significantly between treatments (Figure 2a). Three genera, *Coprococcus* (0.26% vs. 0.65%), *Solobacterium* (0.36% vs. 0.65%), and Family XIII AD3011 group (0.13% vs. 0.38%), were more abundant in the ALA group. In contrast, *Succinivibrio* (2.68% vs. 0.30%), *Mitsuokella* (1.06% vs. 0.28%), and one additional genus were significantly lower in the ALA group.

At 84 days of age, eight genera differed significantly between groups (Figure 2b). The ALA group exhibited higher abundances of *Prevotellaceae* UCG-003 (0.32% vs. 0.68%), *Prevotellaceae* NK3B31 group (1.09% vs. 2.91%), and *Phascolarctobacterium* (0.70% vs. 2.09%). Conversely, *Shuttleworthia* (1.61% vs. 0.08%), *Syntrophococcus* (0.63% vs. 0.07%), *Erysipelotrichaceae* UCG-009 (0.21% vs. 0.02%), and three other genera were significantly lower in the ALA group.

### Oxidative stress, inflammatory status, and plasma IgG concentration

Plasma MDA concentrations did not differ between groups at any sampling point (Table 3). At 56 days of age, leukocyte-derived oxidative (CL-O_2_•®) and inflammatory (FL-OCl̅) markers did not differ between groups. However, by 84 days, CL-O_2_•® levels in the ALA group were approximately 3.5-fold lower than in controls (*p* < 0.05; control: 21.06 ± 4.66 × 10^5^ vs. ALA: 5.97 ± 0.95 × 10^5^). FL-OCl̅ levels also tended to be lower in the ALA group (*p* = 0.052; control: 11.18 ± 1.25 × 10³ vs. ALA: 7.37 ± 1.18 × 10³).

**Table 3:** Oxidative stress and immune biomarkers in piglets fed control and ALA diets at different ages.

Parameter	28-day-old			56-day-old			84-day-old		

	Control	ALA	p-value	Control	ALA	p-value	Control	ALA	p-value
Cl-O2• - (× 10^5^)	NA	NA	NA	17.68 ± 1.28	20.82 ± 2.68	0.589	21.06 ± 4.66	5.97 ± 0.95	0.022
MDA (μM)	3.94 ± 0.47	3.15 ± 0.49	0.275	1.81 ± 0.25	1.35 ± 0.16	0.169	1.75 ± 0.83	3.07 ± 0.63	0.240
Fl-COl- (× 10^3^)	NA	NA	NA	12.25 ± 1.38	13.86 ± 1.10	0.386	11.18 ± 1.25	7.37 ± 1.18	0.052
IgG (mg/mL)	11.5 ± 1.36	11.61 ± 1.96	0.968	10.31 ± 1.38	16.77 ± 4.17	0.228	20.19 ± 3.2	34.66 ± 3.23	0.015

Values are presented as mean ± Standard error. Leukocyte-derived superoxide radical and hypochlorite ion were quantified using a simultaneous chemiluminescence/fluorescence system. Measurements were not performed at 28 days of age due to methodological constraints requiring fresh whole blood. ALA = 5-Aminolevulinic acid, CL-O_2_•^-^ = Chemiluminescence-detected superoxide radical, FL-OCl^-^ = Fluorescence-detected hypochlorite ion, IgG = Immunoglobulin G, MDA = Malondialdehyde, NA = Not analyzed.

Plasma IgG concentrations did not differ at baseline or at 56 days of age. At 84 days, however, the ALA group demonstrated a significantly higher IgG level (1.52-fold increase; p < 0.05; control: 20.19 ± 3.20 mg/mL vs. ALA: 30.66 ± 3.23 mg/mL).

### Correlation between fecal microbiota and oxidative–immune parameters

Because significant group differences in microbiota composition and oxidative/immune markers were observed at 84 days, correlation analyses were performed at this time point.

**Figure 3:**
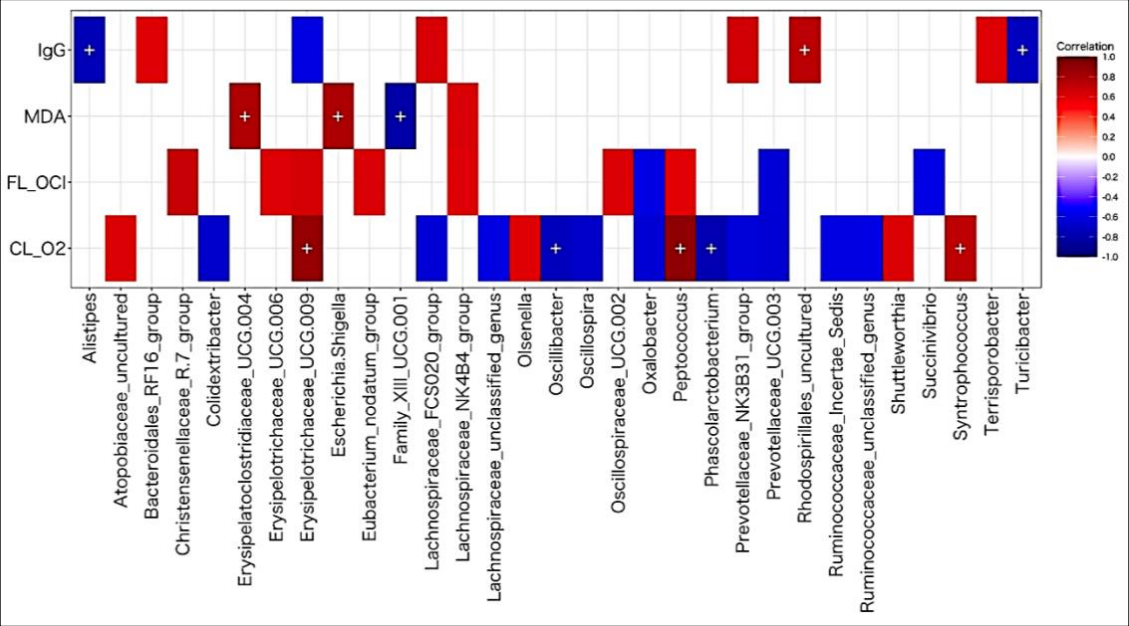
Spearman correlation heatmap showing associations between the relative abundance of fecal bacterial genera and four physiological parameters: immunoglobulin G, malondialdehyde, fluorescence-detected hypochlorite ion, and chemi-luminescence-detected superoxide radical. Red and blue colors indicate significant positive and negative correlations, respectively (p < 0.05), with color intensity reflecting correlation strength. A plus sign (+) denotes p < 0.01.

Seventeen genera exhibited significant correlations with CL-O_2_•®, while 4, 10, and 8 genera were significantly correlated with MDA, FL-OCl̅, and IgG, respectively (Figure 3; Supplementary Table 2). Genera correlated with CL-O_2_•® included *Prevotellaceae* UCG-003, *Prevotellaceae* NK3B31 group, *Phascolarctobacterium*, *Syntrophococcus*, and *Erysipelotrichaceae* UCG-009.

## DISCUSSION

### Overview of findings and novel methodological application

This study evaluated the effects of dietary 5-ALA supplementation on the gut microbiota, oxidative stress, and immune responses of weanling piglets during the post-weaning period. Importantly, this is the first swine study to apply a simultaneous chemiluminescence/fluorescence system to freshly isolated leukocytes, allowing sensitive and real-time detection of oxidative and inflammatory markers. As this work was exploratory, the findings should be interpreted as preliminary yet hypothesis-generating.

### Age-related shifts in microbiota composition

Independent of dietary treatment, fecal microbiota composition (β-diversity) changed markedly between 28 and 84 days of age (p < 0.05). These shifts are consistent with the well-documented microbial transition following weaning, during which Bacteroides decrease due to reduced milk sugar availability, while *Prevotella* increase in response to plant-based dietary substrates [27, 28]. Given these strong age effects, comparisons between the control and ALA groups were performed separately at each sampling point.

At 28 days of age, no meaningful differences in α- or β-diversity or in the abundance of bacterial genera were detected, confirming comparable microbiota profiles between groups at baseline. Similarly, systemic oxidative stress (as indicated by MDA) did not differ between groups at this time.

### Microbiota and physiological changes at 56 days of age

By 56 days, the ALA group exhibited significant differences in β-diversity and in the abundance of several bacterial genera relative to controls, although α-diversity remained unchanged. Notably, the ALA group showed higher levels of *Coprococcus* and *Solobacterium*, genera associated with butyrate and short-chain fatty acids (SCFA) production [29, 30] and previously linked to enhanced growth performance in pigs [31]. This microbial pattern coincided with higher BW in the ALA group at this stage. No group differences in oxidative or inflammatory markers were observed at 56 days.

### Microbiota alterations and oxidative responses at 84 days of age

At 84 days, differences in β-diversity and multiple bacterial genera persisted between the groups. The ALA group demonstrated significantly lower leukocyte-derived CL-O_2_•® levels, indicating reduced oxidative stress. This reduction was accompanied by higher abundances of fiber-degrading or SCFA-associated genera, including *Prevotellaceae* NK3B31 group, *Prevotellaceae* UCG-003, and *Phascolarctobacterium* [29, 32].

These findings align with previous work by Zhang *et al*. [33], who reported decreases in *Prevotellaceae* NK3B31 and UCG-003 under chronic oxidative stress and an increase in *Syntrophococcus*, a genus reduced by 5-ALA in the present study. Here, several of these genera showed significant correlations with CL-O_2_•®, suggesting that modulation of the gut microbiota may influence leukocyte oxidative status.

### Differential sensitivity of systemic vs leukocyte oxidative markers

MDA, a systemic oxidative stress marker reflecting lipid peroxidation in major organs [34], did not differ between groups. In contrast, leukocyte-specific oxidative responses were clearly modulated by 5-ALA. Because leukocytes constitute 70%–80% of immune cell populations within the gut-associated immune system [35], CL-O_2_•® may more directly reflect microbiota–immune interactions than MDA.

Zhang *et al*. [33] similarly observed stronger correlations between microbial genera and oxidative markers in local tissues (liver, jejunum, ileum) than with MDA. Thus, CL-O_2_•® may be a more sensitive indicator of microbially associated oxidative modulation.

### Immunomodulatory effects of 5-ALA

At 84 days, the ALA group exhibited lower FL-OCl̅ values and significantly higher plasma IgG concentrations, indicating attenuation of inflammation and enhancement of humoral immunity. These findings support earlier reports demonstrating immunostimulatory effects of 5-ALA in pigs [10]. Importantly, leukocyte counts did not differ between groups, suggesting that differences in oxidative and inflammatory markers reflect altered leukocyte activation states rather than variations in cell numbers.

Some bacterial genera correlated with CL-O_2_•® also correlated with IgG or FL-OCl̅, while others uniquely associated with immune markers alone. This pattern indicates that changes in microbial composition may contribute to immune alterations but that oxidative and immune pathways are not entirely overlapping.

### Potential mechanisms underlying 5-ALA effects

The mechanisms linking 5-ALA to microbiota modulation remain uncertain. Pharmacokinetic data indicate that orally ingested 5-ALA is absorbed primarily in the liver, small intestine, and kidneys, with limited distribution to the large intestine [36]. Thus, direct utilization of 5-ALA by fecal bacteria is unlikely. Instead, microbiota changes may arise indirectly through 5-ALA–mediated improvements in iron metabolism [8, 37], mitochondrial function [3, 38], or intestinal physiology.

Because microbiota differences emerged at 56 days, prior to significant changes in oxidative and immune markers at 84 days, it is plausible that 5-ALA exerts initial effects at the gut level, particularly given that approximately 20% of ingested 5-ALA is retained within the small intestine. Altered nutrient flow or redox conditions in the intestine could subsequently reshape microbial communities. Future studies incorporating small intestinal functional assessments and microbial metabolite (e.g., SCFA) profiling are needed to clarify these pathways.

### Limitations

This study has several limitations inherent to its exploratory design. First, the sample size was relatively small, which may limit the statistical power and generalizability of the findings. Second, the panel of oxidative and immune markers assessed was narrower than that used in comparable studies, and no tissue-level evaluations, such as inflammatory or oxidative assessments in the small intestine or liver, were performed, as the primary focus was on fecal microbiota. Third, microbial metabolites, including SCFAs, were not measured, restricting the ability to infer the functional metabolic consequences of the observed microbiota shifts. To address these limitations, future research should employ larger cohorts, extended monitoring periods, and a comprehensive multi-omics framework (e.g., metagenomics, metabolomics, transcriptomics). Such approaches will help elucidate the mechanistic links among gut microbiota, microbial metabolites, oxidative stress, and immune responses across multiple tissues, and clarify functional intestinal changes associated with 5-ALA supplementation.

## CONCLUSION

This exploratory study demonstrated that dietary supplementation with 5-aminolevulinic acid (5-ALA; 20 mg/kg feed for 8 weeks) substantially modulated the gut microbiota of weanling piglets and improved indicators of oxidative and immune function during the post-weaning period. Key findings include (i) significant alterations in β-diversity at 56 and 84 days of age, (ii) increased abundances of beneficial SCFA-associated genera such as *Coprococcus*, *Prevotellaceae* UCG-003, *Prevotellaceae* NK3B31 group, and *Phascolarctobacterium*, (iii) a ~3.5-fold reduction in leukocyte-derived superoxide (CL-O_2_•®) in 5-ALA–supplemented pigs at 84 days, and (iv) a 1.5-fold elevation in plasma IgG concentration at the same time point. Together, these results suggest that 5-ALA may strengthen the gut–immune axis through microbial modulation and support redox balance and immune responsiveness during a physiologically vulnerable developmental window.

From a practical standpoint, 5-ALA has potential as a functional feed additive to improve piglet resilience during weaning, an important period characterized by microbial instability, oxidative stress, and heightened disease susceptibility. Enhancing microbial communities associated with SCFA production and reducing leukocyte oxidative burden could translate into better health outcomes under commercial production conditions.

A major strength of this study was the integration of microbiome profiling with real-time measurements of leukocyte oxidative and inflammatory responses using a novel chemiluminescence/fluorescence detection platform in swine research. This approach enabled sensitive detection of functional immune shifts in parallel with microbial changes.

Future research should expand these findings by incorporating larger sample sizes, multiple dosing strategies, longitudinal monitoring beyond eight weeks, and multi-omics approaches, including SCFA quantification, intestinal transcriptomics, metabolomics, and tissue-level oxidative assessments. Such studies will help clarify causal pathways and determine whether 5-ALA can provide consistent, dose-dependent benefits in pig production systems.

In conclusion, the present work provides promising preliminary evidence that 5-ALA supplementation beneficially shapes the fecal microbiota and enhances oxidative and immune status in weanling piglets. While confirmatory studies are required, the results highlight the potential of 5-ALA as a biological modulator to support piglet health, robustness, and productivity during early-life transitions.

## DATA AVAILABILITY

All the generated data are included in the manuscript. The sequence data (Fastq files) have been deposited in the Sequence Read Archive (SRA) under accession number PRJNA1271458.

## AUTHORS’ CONTRIBUTIONS

ST, TT, and RI: Conceptualization. KK (Kiyonori Kawasaki), TT, and RI: Methodology. SI and HM: Formal analysis. SI and KK (Kiyonori Kawasaki), KY, and KK (Kimiko Kazumura): Investigation and data curation. SI and HM: Data curation. SI: Writing—original draft preparation. HM and RI: Writing—review and editing. SI, HM, and RI: Visualization. All authors have read and approved the final version of the manuscript.
